# Prognostic significance of APACHE II score and plasma suPAR in Chinese patients with sepsis: a prospective observational study

**DOI:** 10.1186/s12871-016-0212-3

**Published:** 2016-07-29

**Authors:** Xuan Liu, Yong Shen, Zhihua Li, Aihua Fei, Hairong Wang, Qinmin Ge, Shuming Pan

**Affiliations:** Department of Emergency, Xinhua Hospital, Shanghai Jiaotong University School of Medicine, 1665 Kongjiang Road, Shanghai, 200092 China

**Keywords:** Sepsis, Risk stratification, Soluble urokinase plasminogen activator receptor (suPAR), Acute Physiology and Chronic Health Evaluation II (APACHE II)

## Abstract

**Background:**

Timely risk stratification is the key strategy to improve prognosis of patients with sepsis. Previous study has proposed to develop a powerful risk assessment rule by the combination of Acute Physiology and Chronic Health Evaluation II (APACHE II) score and plasma soluble urokinase plasminogen activator receptor (suPAR). That reaffirmation of suPAR as a prognostic marker in Chinese patients with severe sepsis is the aim of the study.

**Methods:**

A total of 137 consecutive Chinese patients with sepsis were enrolled in a prospective study cohort. Demographic and clinical characteristics, conventional risk factors and important laboratory data were prospectively recorded. Sequential plasma suPAR concentrations were measured by an enzymeimmunoabsorbent assay on days 1, 3, and 7 after admission to the intensive care unit (ICU). Receiver operating characteristic (ROC) curves and Cox regression analysis were used to examine the performance of suPAR in developing a rule for risk stratification.

**Results:**

The results showed that plasma suPAR concentrations remained relatively stable within survivors and non-survivors during the first week of disease course. Regression analysis indicated that APACHE II ≥15 and suPAR ≥10.82 ng/mL were independently associated with unfavorable outcome. With the above cutoffs of APACHE II and suPAR, strata of disease severity were determined. The mortality of each stratum differed significantly from the others.

**Conclusions:**

Combination of APACHE II score and suPAR may supply the powerful prognostic utility for the mortality of sepsis.

## Background

The incidence of sepsis in adults has been increasing, with severe sepsis and septic shock remaining among the major causes of death worldwide [1]. Despite the mortality is on a declining trend in recent years [2], low awareness, late recognition, and improper treatment are still common [3].

One of the fundamental principles for the appropriate management of sepsis is timely discrimination of the patients at high risk for death [4]. This is generally dependent on the application of score systems and plasma biomarkers. Although the well-recognized score is the Acute Physiology and Chronic Health Evaluation II (APACHE II), APACHE II score has some potential pitfalls that may lead to inaccurate evaluation. Take young patients with severe sepsis but without chronic organ dysfunction for instance, the APACHE II score may be relatively low despite the risk for an unfavorable outcome is high [5].

Although various biological markers are widely explored [6–9], only a few have been applied in the clinical practice. The soluble urokinase plasminogen activator receptor (suPAR), which exists in three forms (I-III, II-III and I), is regarded as a novel biomarker of immune system activation [10]. Urokinase plasminogen activator receptor (uPAR) is embedded in the cell membranes of various immunologically active cells and, with its ligand, urokinase plasminogen activator (uPA), takes part in a range of immunologic activities [11]. Upon inflammatory stimulation, uPAR is cleaved from the cell surface by proteases into the soluble form of the receptor-suPAR-which can be assessed in blood, urine, bronchoalveolar lavage, and cerebrospinal fluid [12, 13]. Recent studies have revealed that suPAR may have the ability to predict the mortality of sepsis [14–17]. It is noteworthy that Giamarellos-Bourboulis et al. have proposed a new prognostication rule for predicting the outcome of sepsis by APACHE II score and suPAR [5].

The primary purpose of the present study was to further reaffirm the prediction rule for the mortality in Chinese patients with sepsis by combining APACHE II score and plasma suPAR concentrations.

## Methods

### Study design

This prospective trial involved consecutive Chinese patients with sepsis presenting to the intensive care unit (ICU) of the Department of Emergency, Xinhua Hospital, Shanghai Jiaotong University School of Medicine, from March 2013 to February 2015.

For each patient with suspected infection, a complete diagnostic work-up was performed. The work-up comprised demographic and clinical characteristics, conventional risk factors, and important laboratory data including blood routine examination, microbiological culturing, chest x-ray, and chest or abdominal computed tomography if necessary. Broad spectrum antimicrobial treatment was used within 1 h from the recognition of the septic status.

Patients were eligible if they met the inclusion criteria: (1) age of at least 18 years; (2) sepsis due to one of the following infections: community acquired pneumonia, hospital acquired pneumonia, ventilator-associated pneumonia, acute pyelonephritis, intra-abdominal infection, or primary bacteremia; and (3) blood sampling within 24 h from the presentation of signs of sepsis. Patients affected by advanced cancer or terminal patients with other pathologies were excluded.

All eligible patients were further classified according to standard definitions of sepsis, severe sepsis, and septic shock [18]. More specifically, sepsis was defined as the presence of suspected or confirmed infection together with two or more criteria for a systemic inflammatory response; severe sepsis was defined as sepsis with sepsis-induced organ dysfunction, hypotension or hypoperfusion; septic shock was defined as refractory hypotension or hypoperfusion despite sufficient fluid resuscitation.

### Blood measurements

Venous blood (3 mL) was collected from patients presenting to the ICU (day 1) and repeated on the following day 3 and day 7 after admission. Whole blood was drawn into a centrifuge tube containing EDTA anti-coagulant. After centrifugation at 3,000 g for 10 min at 4 °C, plasma samples were kept frozen at −80 °C until assayed. suPAR was determined in duplicate by a commercial double monoclonal antibody sandwich enzyme immunoassay (suPARnostic® Standard kit; ViroGates A/S, Birkerød, Denmark) in accordance with the instructions of the manufacturer. Every 45 blood samples can be measured within about 4 h. The linearity of this assay is comprised between 2.0 and 15.6 ng/mL, and the total imprecision, expressed as coefficient of variation (CV %), ranges from 2.3 to 6.0 %.

### Study outcomes

Patients who survived were further followed up by telephone calls. The unfavorable outcome of the study was defined as death from any cause within 28 days after admission to the ICU.

### Statistical analysis

Continuous variables were presented as mean values ± standard deviation (SD) or median with interquartile ranges (IQR), while categorical variables were expressed as percentages. The statistical significance of intergroup differences was compared through unpaired Student’s *t*-test or Mann–Whitney *U* test for continuous variables and through Pearson’s *χ* ^*2*^ test for categorical variables.

The following steps were performed to establish a risk stratification rule: First, receiver operating characteristic (ROC) analysis was conducted with baseline levels of APACHE II score and suPAR to determine the prediction sensitivity and specificity of the variables. Second, we used univariate and multivariate Cox regression analyses to calculate hazard ratios (HR) with 95 % confidence intervals (CIs). Third, strata of disease severity were established using the cutoffs of APACHE II score and suPAR. Odds ratios (OR) and 95 % CIs for risk prediction within each stratum were assessed using Mantel and Haenszel statistics. Fourth, mortalities between strata were estimated using the log-rank test.

A two-sided *P* value < 0.05 was considered statistically significant. All analyses were performed by the IBM SPSS Statistics software version 19.0 (SPSS, Chicago, IL, USA).

## Results

### Baseline characteristics of the study population

A total of 137 consecutive patients (51.09 % men; mean age, 69.53 ± 9.28 years) were eligible for enrollment in the study. After the initial evaluation performed in the ICU, patients were divided into three groups according the disease severity: group 1, patients with sepsis (*n* = 56); group 2, those with severe sepsis (*n* = 49); and group 3, those with septic shock (*n* = 32). The baseline clinical and laboratory characteristics of the patients are elaborated in Table 1. The most common locations of infection were lung and urinary tract, and the distribution of locations was similar among the three groups. The commonest isolated pathogens from the study cohort were Gram-negative microorganisms with a predominance of *Escherichia coli*, and blood cultures were positive in 43.80 % of all patients. There was not any difference in pathogen strains among the different groups (Table 1).

**Table 1:** Baseline clinical and laboratory characteristics of the study subjects

	Patient group			
Characteristics	Sepsis	Severe sepsis	Septic shock	*P* value
Demographics and underlying conditions				
Number of patients	56	49	32	-
Males, no. (%)	29(51.79 %)	27(55.10 %)	14(43.75 %)	0.418
Age (years), mean ± SD COPD, no (%) Hypertension, no (%) Diabetes mellitus, no (%)	68.04 ± 9.36 20(35.71 %) 23(41.07 %) 9(16.07 %)	71.39 ± 8.85 24(48.98 %) 23(46.94 %) 9(18.37 %)	69.16 ± 9.73 19(59.38 %) 17(53.13 %) 7(21.88 %)	0.246 0.032* 0.473 0.381
Baseline parameters, mean ± SD				
APACHE II score SOFA score	9.87 ± 3.12 5.26 ± 2.09	12.50 ± 4.75 7.83 ± 2.53	18.34 ± 6.09 11.42 ± 3.74	0.001** 0.002**
SuPAR (ng/mL) PCT (ng/mL) White blood cell count (10^9^/L)	6.58 ± 3.17 6.14 ± 3.54 11.96 ± 2.866	8.62 ± 4.80 11.05 ± 4.60 18.26 ± 3.98	15.97 ± 5.44 27.69 ± 7.28 26.67 ± 7.04	0.001** <0.001** 0.037*
Lactic acid (mmol/L)	1.75 ± 1.16	3.27 ± 1.48	6.03 ± 3.82	0.012*
BUN (mmol/L) Scr (μmol/L) ALT (U/L) AST (U/L) Bilirubin (mg/dL) Platelet (10^9^/L) Plasma glucose (mmol/L) Hemoglobin (g/L)	6.42 ± 3.85 70.32 ± 19.56 31.23 ± 10.27 32.95 ± 11.02 14.60 ± 8.03 154.19 ± 71.52 7.25 ± 4.89 109.84 ± 33.14	9.05 ± 4.13 103.59 ± 31.07 45.62 ± 16.44 60.38 ± 21.37 16.23 ± 9.15 134.08 ± 75.17 8.35 ± 5.18 117.08 ± 20.49	11.91 ± 9.39 154.08 ± 40.87 153.89 ± 48.01 196.35 ± 65.29 20.01 ± 9.66 127.60 ± 65.95 10.02 ± 7.11 118.05 ± 20.65	0.017* 0.021* 0.013* 0.009** 0.694 0.741 0.389 0.436
Pathogen strains, no (%)				0.058
Escherichia coli Klebsiella pneumonia Pseudomonas aeruginosa Acinetobacter baumannii Other Gram-negative bacteria Staphylococcus aureus Enterococcus spp	6(10.71 %) 5(8.93 %) 2(3.57 %) 3(5.36 %) 2(3.57 %) 1(1.79 %) 1(1.79 %)	6(12.24 %) 4(8.16 %) 3(6.12 %) 4(8.16 %) 2(4.08 %) 2(4.08 %) 1(2.04 %)	5 (15.63 %) 4(12.50 %) 3(9.38 %) 3(9.38 %) 1(3.13 %) 2(6.25 %) 0(0.00 %)	- - - - - - -
Site of infection, no (%)				0.713
Lung Urinary tract Abdomen Other	18(32.14 %) 15(26.79 %) 11(19.64 %) 12(21.43 %)	23(46.94 %) 13(26.53 %) 7(14.29 %) 6(12.24 %)	15(46.88 %) 8(25.00 %) 6(18.75 %) 3(9.38 %)	- - - -
Intervention, no (%)				
Mechanical ventilation CRRT Vasopressor usage Study outcome, no (%) 28-day mortality	3(5.36 %) 2(3.57 %) 0(0.00 %) 2(3.57 %)	4(8.16 %) 6(12.24 %) 4(8.16 %) 4(8.16 %)	12(37.50 %) 15(46.88 %) 13(40.63 %) 14(43.75 %)	0.005** 0.004** 0.001** 0.001**

Abbreviations: *COPD* chronic obstructive pulmonary disorder, *APACHE II* Acute Physiology and Chronic Health Evaluation II, *SOFA* sequential organ failure assessment, *suPAR* soluble urokinase plasminogen activator receptor, *PCT* procalcitonin, *BUN* blood urea nitrogen, *Scr* serum creatinine, *ALT* alanine transaminase, *AST* aspartate transaminase, *CRRT* continuous renal replacement therapy

Data are expressed as no. (%), or mean (standard deviation, SD) as appropriate

Significant differences are marked by *(*P* < 0.05) or **(*P* < 0.01)

There were no significantly statistical differences in patients with sepsis compared to those in severe sepsis or septic shock for gender or age. Patients with severe sepsis or septic shock tended to have higher baseline levels of APACHE II score, Sequential Organ Failure Assessment (SOFA) score, suPAR, procalcitonin (PCT) and lactic acid compared with patients with sepsis. In addition, there were 19 patients (13.87 %) receiving mechanical ventilation treatment, 23 patients (16.79 %) receiving continuous renal replacement therapy, and 17 patients (12.41 %) receiving vasopressor support. There were significant differences in the proportion of patients receiving mechanical ventilation, continuous renal replacement therapy or vasopressor support among the three groups (Table 1).

### Kinetics of suPAR

**Fig. 1:**
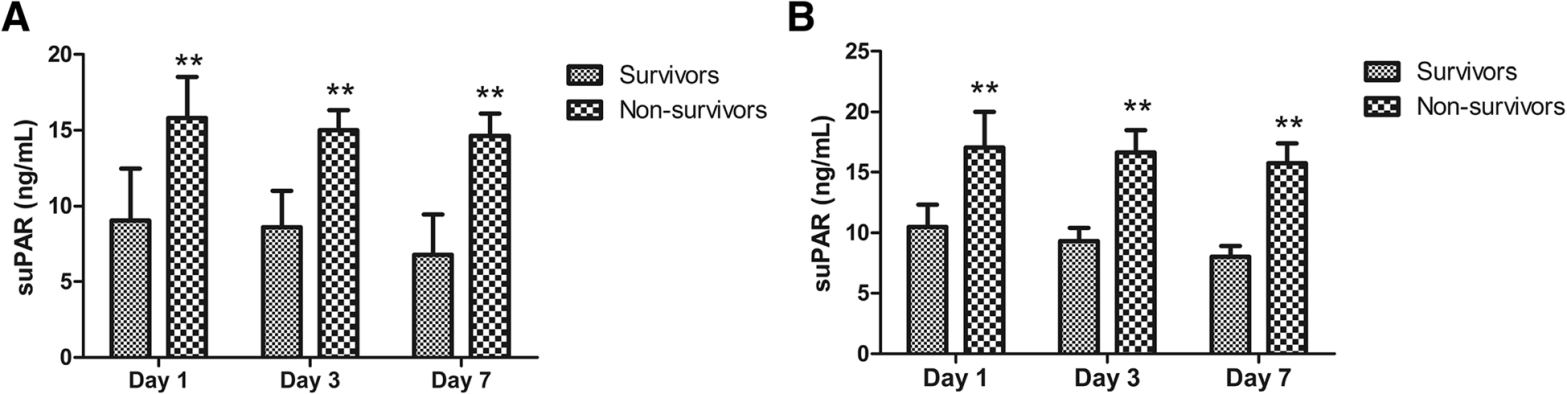
Plasma suPAR concentrations among survivors and non-survivors during the course of 7 days. **a** Plasma suPAR concentrations among 117 survivors and 20 non-survivors from all the patients. **b** Plasma suPAR concentrations among 18 survivors and 14 non-survivors from the patients with septic shock. Values are expressed as mean ± SD. **P < 0.01 between survivors and non-survivors at the indicated day of sampling. suPAR, soluble urokinase plasminogen activator receptor

Among the enrolled patients, a total of 117 patients survived and 20 died. As shown in Fig. 1a, patients who died had significantly higher suPAR concentrations (15.82 ± 2.72 ng/mL) on admission in comparison with the survivors (9.04 ± 3.41 ng/mL, *P* < 0.01). To investigate whether plasma suPAR concentrations remain constant over time, serial plasma determinations were further conducted on day 3 and day 7 after admission. At each indicated day of sampling, plasma suPAR concentrations were markedly higher among non-survivors than among survivors. Plasma suPAR concentrations remained stable separately within survivors and within non-survivors during the first week of the disease course. In addition, in the septic shock group 14 patients died and 18 survived. These non-survivors had significantly higher suPAR concentrations (17.05 ± 2.96 ng/mL) on admission when compared with the survivors (10.48 ± 1.86 ng/mL, *P* < 0.01) in the septic shock group (Fig. 1b).

### Value of indicators in predicting poor outcome

**Fig. 2:**
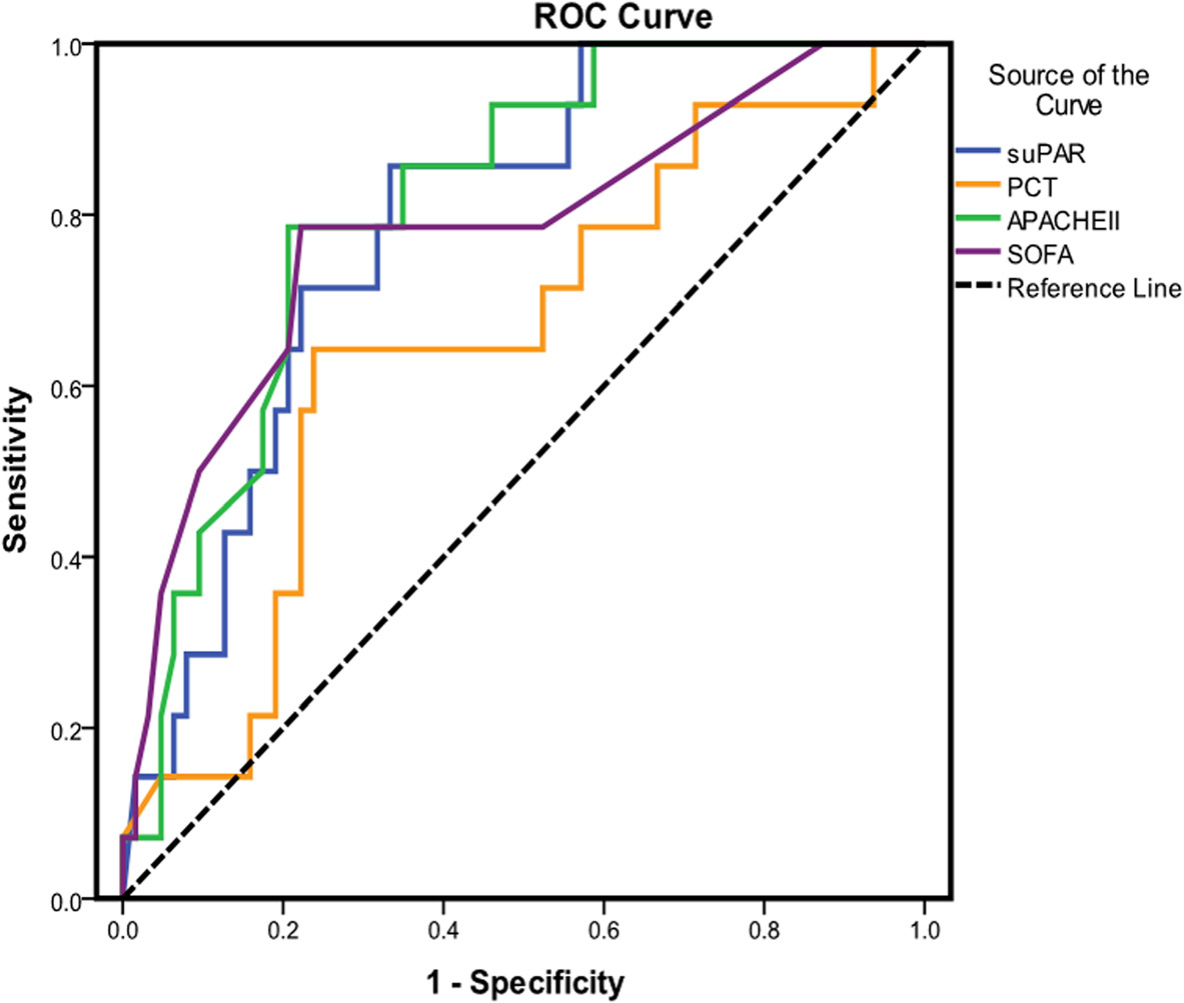
Receiver operating characteristic (ROC) curves of suPAR, PCT, APACHE II score, and SOFA score on day 1. suPAR had a strong power for predicting unfavorable outcome as suggested by area under the curve (AUC) of 0.788 ± 0.058, *P* = 0.001. suPAR, soluble urokinase plasminogen activator receptor; PCT, procalcitonin; APACHE II, Acute Physiology and Chronic Health Evaluation II; SOFA, Sequential Organ Failure Assessment

ROC analysis was constructed to examine the performance of indicators as predictors of poor outcome, and the area under the curve (AUC) for each indicator was calculated, respectively. The AUC, optimal cutoff value, sensitivity and specificity of each indicator are presented in Table 2. ROC curves indicated that suPAR had a strong power for predicting unfavorable outcome as suggested by AUC of 0.788 ± 0.058, which was less than that of APACHE II score (0.813 ± 0.055, *P* < 0.05) but greater than that of SOFA score (0.779 ± 0.075, *P* < 0.05) and PCT (0.651 ± 0.081, *P* < 0.01) (Fig. 2). Coordinate points of ROCs indicated that an APACHE II score of at least 15 as a cutoff had a specificity of greater than 70 % to predict death and suPAR of at least 10.82 ng/mL showed a specificity of greater than 70 % to predict death.

**Table 2:** Performance of variables in predicting unfavorable outcome

Variables	AUC ROC	*P* value	Cutoff value	Sensitivity (%)	Specificity (%)
APACHE II score SOFA score	0.813 ± 0.055 0.779 ± 0.075	<0.001** <0.001**	≥15.00 ≥8.50	89.6 80.7	74.8 72.1
suPAR	0.788 ± 0.058	<0.001**	≥10.82	84.9	77.6
PCT	0.651 ± 0.081	0.078	≥24.97	57.5	69.2

Abbreviations: *AUC ROC* area under the receiver operating characteristic curve, *APACHE II* Acute Physiology and Chronic Health Evaluation II, *SOFA* sequential organ failure assessment, *suPAR* soluble urokinase plasminogen activator receptor, *PCT* procalcitonin

Significant differences are marked by **(*P* < 0.01)

**Fig. 3:**
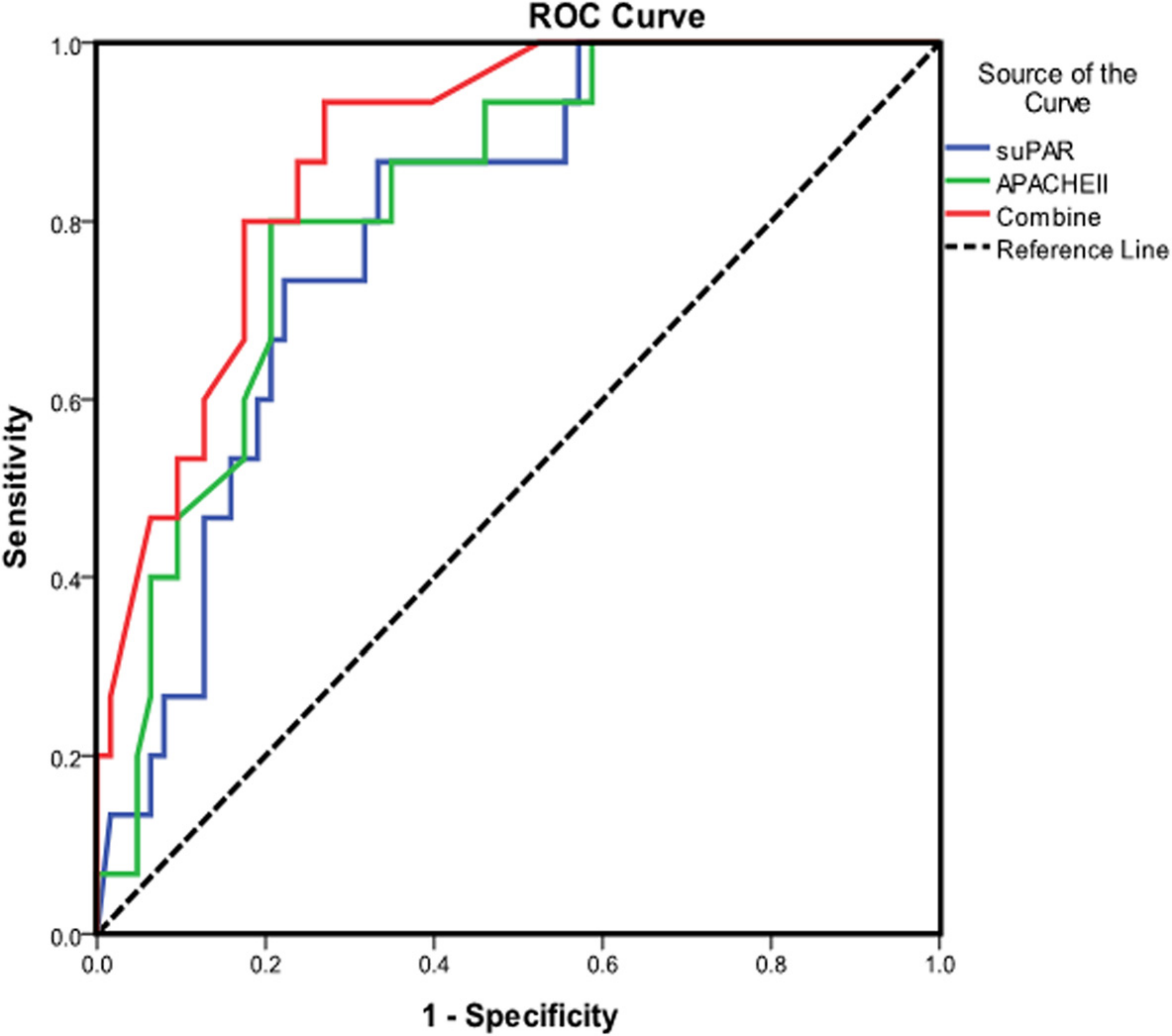
Receiver operating characteristic (ROC) curves of suPAR, APACHE II score, and their combination on day 1. The combination of suPAR and APACHE II score had a strong power for predicting unfavorable outcome as suggested by area under the curve (AUC) of 0.878 ± 0.042, *P* = 0.000. suPAR, soluble urokinase plasminogen activator receptor; APACHE II, Acute Physiology and Chronic Health Evaluation II

Furthermore, ROC analysis of the combination of APACHE II score and suPAR was further performed. We found that the AUCs were greater for the combination of APACHE II score and suPAR (0.878 ± 0.042) than for the single APACHE II score or single suPAR (Fig. 3), demonstrating that combination of APACHE II score and suPAR may supply the more powerful prognostic utility for the mortality of sepsis.

### Univariate Cox regression analysis

We performed univariate Cox regression analysis to examine the associations of each variable with unfavorable outcome and calculated the standardized regression coefficient (**β**) and the HR for each variable. As shown in Table 3, baseline APACHE II score had the greatest absolute value of standardized **β** value (0.2457). The absolute value of standardized **β** value for suPAR was 0.1482 and the unadjusted HR was 2.18 (95 % confidence interval [CI], 1.77-2.95, *P* = 0.000), indicating that suPAR had a power for predicting unfavorable outcome.

**Table 3:** Predictors of unfavorable outcome by univariate Cox regression analysis

Variables	Standard β	Hazard ratio	95 % CI	*P* value
Age Gender Lactic acid BUN Scr	0.0683 0.0815 0.0368 0.0240 0.0217	1.07 1.34 1.02 1.01 1.00	0.84–1.50 0.91–1.83 0.82–1.15 0.57–1.12 0.53–1.04	0.634 0.759 0.332 0.458 0.465
APACHE II score SOFA score	0.2457 0.1243	3.01 1.68	2.26–4.69 1.14–2.39	<0.001** <0.001**
SuPAR PCT	0.1482 0.0891	2.18 1.67	1.77–2.95 1.42–2.08	<0.001** 0.024*

Abbreviations: *BUN* blood urea nitrogen, *Scr* serum creatinine, *APACHE II* Acute Physiology and Chronic Health Evaluation II, *SOFA* sequential organ failure assessment, *suPAR* soluble urokinase plasminogen activator receptor, *PCT* procalcitonin, *CI* confidence interval

The hazard ratio indicates the risk of obtaining unfavorable outcome

Significant differences are marked by *(*P* < 0.05) or **(*P* < 0.01)

### Multivariate Cox regression analysis

A multivariate Cox regression analysis was conducted using a forward step-wise manner to determine a novel risk stratification rule. All the observed baseline parameters like age, gender, lactic acid, blood urea nitrogen, serum creatinine, APACHE II score, SOFA score, suPAR and PCT were included in the prediction model when advent of death was set as the dependent variable. The results are shown in Table 4. According to this analysis, APACHE II score of at least 15 and plasma suPAR concentrations of at least 10.82 ng/mL were the independent predictors which entered the equation, demonstrating that these above defined cutoff values may be safely used to create a stratification rule for evaluating unfavorable outcome in sepsis.

**Table 4:** Independent predictors of unfavorable outcome by multivariate Cox regression analysis

Variables	Standard β	Hazard ratio	95 % CI	*P* value
APACHE II score	0.2743	3.57	2.38–4.40	<0.001**
SuPAR	0.1530	2.26	1.94–2.87	<0.001**

Abbreviations: *APACHE II* Acute Physiology and Chronic Health Evaluation II, *suPAR* soluble urokinase plasminogen activator receptor, *CI* confidence interval

The hazard ratio indicates the risk of obtaining unfavorable outcome

Significant differences are marked by **(*P* < 0.01)

The prognostic significance of suPAR was further confirmed after the risk stratification rule was generated (Table 5). More precisely, OR for death with suPAR of at least 10.82 ng/mL among patients with an APACHE II score of less than 15 was 4.72; OR was 2.04 with suPAR of at least 10.82 ng/mL among patients with an APACHE II score of at least 15. The calculated ORs were significantly different, demonstrating that APACHE II score and suPAR were independently associated with the unfavorable outcome and could both be integrated into a risk stratification rule.

**Table 5:** Validation of the novel stratification rule

APACHEII score	suPAR	Survivors, Number (%)	Non-survivors, Number (%)	*P* value	OR (95 % CI)
<15	<10.82	71(98.61 %)	1(1.39 %)	<0.001**	4.72 (3.36–5.81)
	≥10.82	24(88.89 %)	3(11.11 %)		
≥15	<10.82	13(72.22 %)	5(27.78 %)	<0.001**	2.04 (1.85–3.50)
	≥10.82	9(45.00 %)	11(55.00 %)		

Abbreviations: *APACHE II* Acute Physiology and Chronic Health Evaluation II, *suPAR* soluble urokinase plasminogen activator receptor, *OR* odds ratio, *CI* confidence interval

The OR indicates the risk of obtaining unfavorable outcome

Significant differences are marked by **(*P* < 0.01)

### Risk stratification rule of APACHE II score and suPAR

**Fig. 4:**
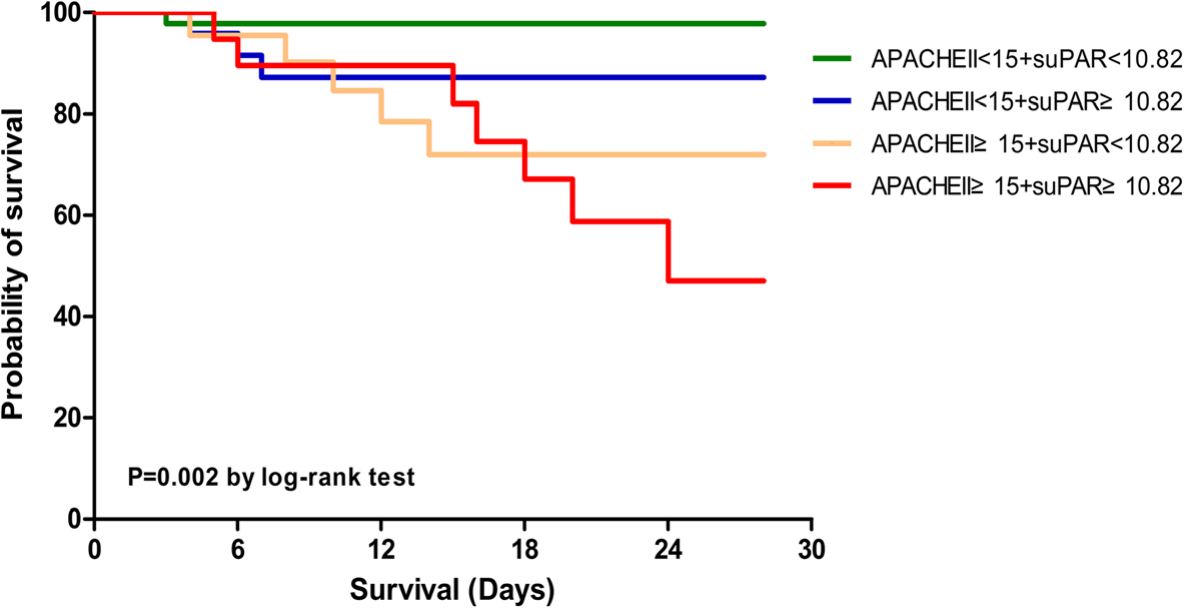
Kaplan-Meier estimates of survival of patients stratified into four strata of severity. Every stratum differed significantly from the others. *P* = 0.002 by the log-rank test within the four defined strata. APACHE II, Acute Physiology and Chronic Health Evaluation II; suPAR, soluble urokinase plasminogen activator receptor

On the basis of the above cutoffs of APACHE II score and suPAR, risk stratification rule was determined as follows: (A) patients with an APACHE II score of less than 15 and suPAR of less than 10.82 ng/mL, (B) patients with an APACHE II score of less than 15 and suPAR of at least 10.82 ng/mL, (C) patients with an APACHE II score of at least 15 and suPAR of less than 10.82 ng/mL, and (D) patients with an APACHE II score of at least 15 and suPAR of at least 10.82 ng/mL. There were 72, 27, 18, and 20 patients in each stratum, with respective mortalities of 1.39 % (*n* = 1), 11.11 % (*n* = 3), 27.78 % (*n* = 5), and 55.0 % (*n* = 11). As show in Fig. 4, each stratum differed significantly from the others (*P* = 0.002 by the log-rank test within the defined strata). This prediction score corresponded to different grades of disease severity, therefore patients with severe sepsis/septic shock tended to have score levels (C) and (D) when patients with sepsis tended to have score levels (A) and (B).

## Discussion

Undoubtedly, APACHE II score has been advocated as the gold standard for risk evaluation in critically ill patients [19]. Nevertheless, a growing body of evidence has suggested that the score may supply inaccurate information in the certain patients, such as disproportionately high scores in patients who are loss of consciousness [20]. This translates into a real-world context in which the efficacy of APACHE II score to predict death is not as powerful as clinicians would consider.

To our knowledge, previous study conducted by Giamarellos-Bourboulis et al. has proposed a new prognostication rule for predicting the outcome of sepsis by APACHE II score and suPAR [5]. Our study was to further reaffirm the risk stratification system for Chinese patients with sepsis by combining APACHE II score and plasma suPAR concentrations. There were some differences between our study and Giamarellos-Bourboulis’s study. First, the enrolled patients of the two studies were from different ethnic groups. We enrolled Asian populations (Chinese origin), while Giamarellos-Bourboulis et al. mainly enrolled the European populations. Second, the cutoffs of APACHE II and suPAR which were used to determine the strata of disease severity were not uniformly the same. Specifically, our study indicated that APACHE II ≥15 and suPAR ≥10.82 ng/mL were independently associated with unfavorable outcome while Giamarellos-Bourboulis’s study showed that APACHE II ≥17 and suPAR ≥12 ng/ml were the optimal cutoffs. Third, we constructed ROC analysis and calculated the AUC to compare the performance of suPAR, PCT, APACHE II score, and SOFA score as predictors of poor outcome. We found that suPAR had a strong power for predicting unfavorable outcome as suggested by AUC of 0.788 ± 0.058, which was less than that of APACHE II score (0.813 ± 0.055) but greater than that of SOFA score (0.779 ± 0.075) and PCT (0.651 ± 0.081). However, Giamarellos-Bourboulis et al. just conducted ROC analysis with suPAR and APACHE II score as independent variables to predict unfavorable outcome. Taken together, given enrolled patients were from different ethnic groups, our study may further confirm the preliminary conclusion that a prediction rule with four levels of risk in sepsis based on APACHE II score and suPAR was proposed.

Similar to the findings of a previous clinical trial concerning plasma suPAR measurement [15], our study clearly showed that suPAR concentrations were relatively stable in the systemic circulation in both survivors and non-survivors during the first week of the disease course. Therefore, we infer that the validity of the developed prognostication score remains constant even if suPAR is not measured during the very first days after ICU admission due to the stability of suPAR concentrations over the disease course. These findings were comparable to other diseases including chronic obstructive pulmonary disease (COPD) [21] or acute respiratory distress syndrome (ARDS) [22], in which suPAR was regarded as an independent predictor for unfavorable outcomes.

Severe sepsis has a reported annual incidence in adults of up to 300 cases per 100,000 population [23, 24]. Affected patients have high mortalities, complications, and resource utilization. Although figures have improved in the recent years [2, 3], the risk for death remains high [25]. Consequently, improving outcome may be a daunting work. One of pivotal measures is to identify the septic patients with poor prognosis rapidly [4]. Our study suggested one composite rule for determining patients with sepsis at high risk on the basis of APACHE II score and plasma suPAR concentrations. Actually, the measurement procedure is so simple that we can complete the measurement for every 45 patient samples within about 4 h. The price for the measurement is also relatively reasonable and we only spend 6,000 CNY (about 924 USD) on evaluating 45 patient samples, that means we need spend about 133 CNY (20 USD) on measuring one patient sample. Undoubtedly, the suPAR measurement is relatively cost-effective. Therefore, given the simple and inexpensive measurement, the combination of APACHE II score and plasma suPAR concentrations may contribute to intensive care management in the septic patients properly.

Currently, evidence has suggested that the value of single scoring system as a standard of clinical decision-making in septic patients is questionable. APACHE II score is likely to recognize either low-risk patients or very-high-risk patients, but not these patients between the two extremes [20]. The proposed risk stratification rule fulfills this need because it discriminates not only patients lying at one of the two extremes - strata (A) and (D) - but also patients with moderate disease severity, namely patients with an APACHE II score of less than 15 and suPAR of at least 10.82 ng/mL or patients with an APACHE II score of at least 15 and suPAR of less than 10.82 ng/mL, who belong to strata (B) and (C), respectively.

## Conclusions

In summary, combination of APACHE II score and suPAR may supply the powerful prognostic utility for the mortality of sepsis. Our findings suggest that incorporating suPAR into APACHE II score as a composite risk stratification rule for sepsis is worth considering.

## Abbreviations

APACHE II, Acute Physiology and Chronic Health Evaluation II; ARDS, acute respiratory distress syndrome; AUC, area under the curve; CI, confidence interval; COPD, chronic obstructive pulmonary disease; ICU, intensive care unit; IQR, interquartile ranges; OR, odds ratio; PCT, procalcitonin; ROC, receiver operating characteristic; SD, standard deviation; SOFA, sequential organ failure assessment; suPAR, soluble urokinase plasminogen activator receptor; uPA, urokinase plasminogen activator; uPAR, urokinase plasminogen activator receptor
